# The 12^th^ century bronze doors of Barisanus of Trani in Trani, Ravello and Monreale

**DOI:** 10.1371/journal.pone.0319697

**Published:** 2025-03-26

**Authors:** Marianne Mödlinger, Bastian Asmus, Martin Fera, Judith Utz, Giorgia Ghiara

**Affiliations:** 1 IMAREAL, Paris Lodron University Salzburg, Krems/Donau, Austria; 2 Labor für Archäometallurgie, Herbolzheim, Germany; 3 Universität Wien, Institut für Urgeschichte und Historische Archäologie, Vienna, Austria; 4 DISAT, Politecnico di Torino, Torino, Italy; Universidad de Sevilla, SPAIN

## Abstract

The bronze and brass doors of the 11th and 12th centuries represent the largest group of medieval monumental bronzes still preserved today in Europe. Of the less than 30 doors that have survived, three, the doors of Trani, Ravello and Monreale, were made by Barisanus of Trani. In this work, we present, for the first time, chemical analyses of the metal parts of all the doors and an in-depth study of their production, showing how a detailed observation of the casting characteristics provides information on the chronological order of the doors studied. This paper contributes to the ongoing discussion in art history regarding the chronological order of the construction of the doors. Moreover, the paper demonstrates the potential of scaled orthometric images as a basis for art historical and production-related questions.

## 1. Introduction

**Fig 1:**
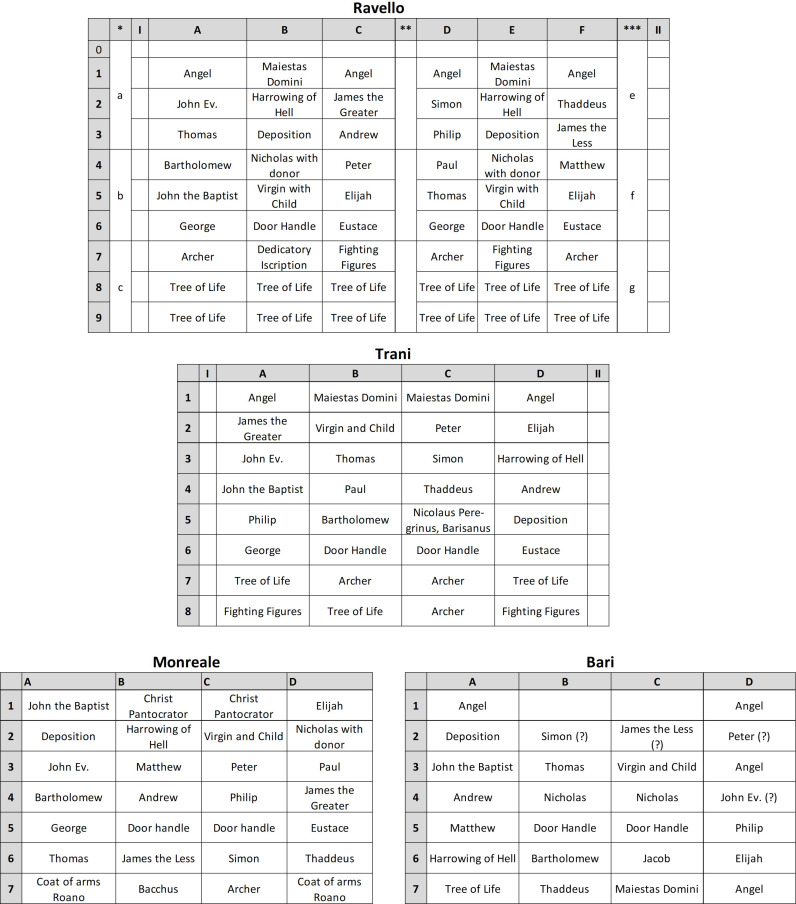
Panel scheme of the four doors from Barisanus of Trani.

**Fig 2:**
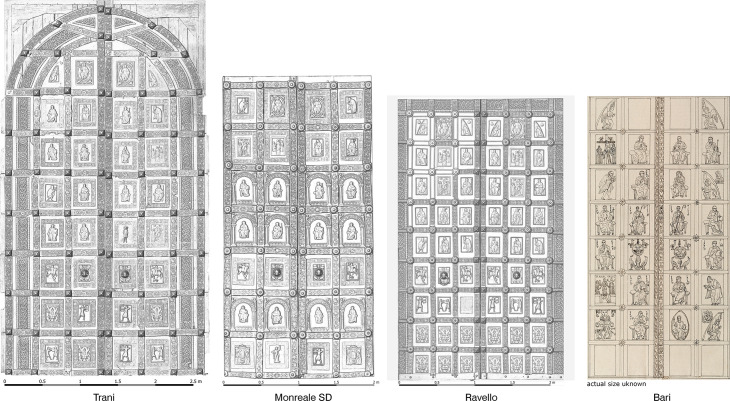
The four doors from Barisanus of Trani. Relief visualization of digital surface models (DSMs). High-resolution images of every door are provided in the supplementary material (S1-S4 Figs): a) Ravello; b) Trani; c) Monreale; d) Bari (design of I. Aveta. Reproduction after: BNF, Paris: plate P62820, Vol. VB-132 (A,1)-FOL; © BNF).

Barisanus of Trani’s workshop was one of the most productive bronze workshops of the High Middle Ages (1000–1300 CE), of which we still have a considerable amount of work preserved today. The workshop produced the doors of the cathedrals of Trani (S. Maria Assunta e S. Nicola Pellegrino, main portal west façade), Ravello (San Pantaleone, main portal west façade) and Monreale (side portal, north façade), which are still preserved today [1–4]. Moreover, there was a door for San Nicola in Bari, although it no longer exists. A drawing in the Bibliothèque Nationale de France by Ignazio Aveta depicts its condition in 1813 (Figs 1 and 2) [5,6]. The actual role of Barisanus in the production of these doors is still not clear, even though he is noted as the “maker” of the doors of Trani and Monreale.

The Barisanus doors are structurally based on bronze doors imported from Constantinople, which can be found in various locations, including Amalfi, Montecassino, Monte Sant’Angelo, Rome, Atrani, Salerno, and Venice [7,8]. Like their predecessors, the Barisanus doors consist of multiple flat bronze panels attached to a wooden support. The panels are fixed to the wooden base via a frame system and nails, with specially designed knobs at the intersections. Unlike the Byzantine doors, the Barisanus doors were designed using engravings rather than niello or silver inlays. In contrast to Mende’s observation [4], no silver inlay could be confirmed on the Ravello B4 panel (Nicholas with donor). Additionally, the figures produced by the Barisanus workshop were in flat relief, in contrast to the completely flat Byzantine figures.

### 1.1 Iconography

The iconographic program of the four known doors is similar. There are only two narrative scenes: the Descent from the Cross and the Anastasis. All other panels show frontal, mostly seated saints, prophets, and apostles, the Maiestas Domini, and the Virgin with Child. Two lionhead door pullers were placed on separate panels, and there are also several archers and dueling groups and animal and plant motifs. The lower section of the door exclusively contains the latter motifs, whereas the Maiestas Domini is situated at the center and top, in Trani and Ravello framed by angels. The four doors depict the same saints repeatedly. The Ravello door has more panels (54) than the Trani (32) and Monreale (28) doors do, which may explain why some saints are duplicated. According to Aveta’s drawing, the Bari door had 32 panels—at least at the beginning of the 19th century—similar to those in Trani. Notably, St. Nicholas, with the donor in proskynesis, is depicted twice here, and the door features four panels of angels. Both features indicate that the Bari door originally had six registers (as did Ravello), unlike the door depicted in the drawing [5]. It is unclear whether this door once adorned the main portal of San Nicola, which features an arch presumably built in the second half of the 12th century [9]. The doubling of the St. Nicholas panels, likely arranged in the center of each wing, suggests this possibility. Regarding Bari, it is noteworthy that Aveta identifies one depiction as St James (panel C6), although St James does not appear on the other known Barisanus doors. For all doors, the original panel arrangement is not always evident due to centuries of panel swapping [2,10]. Iconographically, all Barisanus doors depict a redemption theme that is typical for doors. This is also evident in the words written in the open book of Jesus in the representation of the Maiestas Domini on every door: *Ego sum via et veritas et vita* (John 14,6) [4].

The iconographies of the three Barisanus doors are not only very similar, but the panels also exhibit numerous formal parallels. Art historians have long suspected that the workshop used the same models for casting individual panels, thus saving a considerable amount of time [4]. Differences can be identified only in the depictions and inscriptions of artists and donors. In Monreale, the caster Barisanus is depicted in a proskynesis pose in front of St. Nicholas of Myra. Above St. Nicholas, there is an engraved inscription that reads ‘*Barisanus Tranensis me fecit*’ [1]. In Trani, there is an artist’s depiction at the feet of St. Nicholas Peregrinus, the patron saint of Trani, to whom the cathedral is dedicated. There is also an inscription identifying Barisanus: ‘*Barisanus Tranensis*’. Additionally, on the door of Ravello, there is a reclining figure at the feet of St. Nicholas of Bari. However, in this case, it depicts the donor of the door, Sergio Muscetola, who is also identified by an inscription. Although Barisanus did not sign this door, it is certain that the Ravello door was made in the same workshop because of its great formal similarity to the doors of Monreale and Trani.

### 1.2 Dating

The identity of the donor who gifted the door in Ravello is solely discernible from the depiction on the door and a lengthy inscription. The inscription also specifies the year the door was created and reads as follows:

‘*Anno millesimo centesimo septuagesimo nono incarnacionis Jesu XPO domino nostro memento domine famulo tuo Sergio Musetulae et uxori sue Sicilgaudae et filiis suis Mauro et Iohannes et filia sua Anna quot istam portam facere agit ad honorem Dei et sancte Mariae virginis*’

[In the 1179th year after the birth of our Lord Jesus Christ. Remember, O Lord, your servant Sergius Musetula and his wife Sicligauda and his sons Maurus and John and his daughter Anna, because he had this door made for the glory of God and the Blessed Virgin Mary].

Regarding the Monreale door, it is likely that the Barisanus door dates back to the same time as the main portal of Bonannus of Pisa, or possibly even earlier, as western facades are typically the most recent component. An inscription on the bronze door of Bonannus of Pisa indicates that the door was completed in 1185/86 (‘*Anno domini MCLXXXVI indictione III Bonanus civis Pisanus, me fecit*’) [4]. The Barisanus door can thus be dated between 1174, which corresponds to the period of the cathedral’s foundation by King William II (1166–1189), and 1186. The precise dating of the Trani door remains unknown.

Art historians have traditionally considered Ravello to be the oldest of the three surviving Barisanus doors owing to its minimal ornamentation framing the figures on the panels. The Trani door enhances this ornamental program, and the Monreale door additionally shows arcades emphasizing saints [1,2,4,10]. Importantly, the client’s preferences or the available space may have influenced the level of decoration on the doors, as seen in the cases of Monreale and Trani. The doors in Monreale may have been more richly decorated because of the client’s wishes, whereas the Trani door may have had more ornamental borders because of the availability of more space in the panels.

The fact that Barisanus of Trani was an Apulian artist who apparently made doors for Trani and Bari suggests instead that these two doors belong to the earlier ones. It was only later that the fame of the bronze caster may have made him known beyond the region. The Parisian drawing of the Bari door suggests that it must have been located on the important building of the Basilica of San Nicola. There, it may have adorned either the monumental main portal or two smaller portals [5,6].

### 1.3 Combining art history methods with scientific and digital methods

The dating and chronology of the doors could be more accurately determined by analyzing their surfaces. If all the doors were indeed made using the same molds or copied from one another, they would have become more worn over time unless the models were reworked. Ursula Mende has already observed that the outlines of the figurative depictions on the panels from Monreale are less distinct, which could suggest that this is the most recent of the surviving doors. [4] Our project successfully used scaled orthomosaics to capture selected motives and conduct comparative metric analyses, gaining new insights into the chronology of the three remaining doors. However, a problem quickly became apparent: the state of preservation of the doors, particularly in Monreale, was strongly affected by surface corrosion. As a result, the outlines of the depictions are often distorted by the thick patina. The photographs and analyses of the Monreale doors were taken due to organizational issues prior to the recent restoration of the door by Carlo Usai at the end of 2023.

## 2. State of the art

While detailed chemical analyses of the Ravello and Monreale doors have not yet been published, analyses of the Trani door have already been carried out by G. Secchi [11] and J. Riederer [12]. Unfortunately, the one analysis carried out by Secchi cannot be associated with any specific panel. A similar problem occurs with Riederer’s AAS analyses of drilling samples. The many analyses carried out on different panels of the Trani door cannot be linked to specific panels or sampling locations, which makes these analyses difficult to use.

Restoration work was carried out on all three doors. Concerning Ravello, it is likely that some of the original panels are missing. A description of the door in 1651 stated that it was approximately 35 cm higher. In addition, the earliest restoration report of 1756 stated that one of the panels had been stolen [13]. It is likely that the original (?) wooden base was also replaced at the end of the 18th century with one made of chestnut. In 1871, several other parts appear to be missing; a comparison with a photograph by Salazaro 1871 shows that 31 pieces of metal are no longer extant. Restoration work was carried out after 1887, and the missing pieces were replaced with recent copies. The original metal parts were moved from top to bottom, and the new reconstructed parts were placed mainly on the upper left part of the doors. The last restoration took place between 1989 and 1993 and was carried out by Tudor Dinka. The panels were rearranged approximately 2013, and approximately 880 old (but not necessarily original) iron nails were replaced with steel nails [13].

The doors from Trani also underwent restoration work. During the 18th century, all nails were replaced with newer nails. This happened likely around 1725, as this year was incised on the backside of the wooden door: as shown by the last restoration at the end of the 1990s, the wooden base largely seems to still be the original one from the 12^th^ century, with few additions from the 18^th^ century [14]. Between 1880 and 1894, the metal parts weighing between 18 and 46 kg were unmounted, cleaned with soap and oil inside the church, and mounted again on the previous wooden support. Unfortunately, no information was provided about lost, damaged, or replaced metal parts, apart from the note that “some” were repaired [15] or remade [16]. The wooden base, made of larch and fir, derived most likely from the forests of Sila, so an ICR report from 1951. During restoration works by the ICR 1949-1950, the following treatments were carried out: removal of the previous crude restoration; new sutures, without welding, of the shattered parts; removal of the encrustations; fixing of the patina; new application of the nail heads on a steel rod with a screw nut; and restoration of the rotten parts of the support wood (which is still the larch wood from the 18^th^ century) [1,17]. Between 1989 and 1992, the Trani door was restored again by the Reindell company (Rome), which included the dismantling of the metal parts from the wooden corpus, as well as the cleaning, washing and anticorrosion treatment of the individual metal parts [14]. The work was continued by Soprintendenza between 1998 and 1999 [18]. The panels were mechanically cleaned and washed with sodium sesquicarbonate and distilled water at 60 °C to remove the soluble salts present. Finally, the bronze was treated with corrosion inhibitors and protected with microcrystalline wax. On selected panels (A2, A3, C5, and C8), chemical analyses were carried out (ICP AES). The copper panels inserted during the 1950s were removed and replaced with stainless steel plates. The added bronze was removed and not replaced. All the nails, both the original and those added in later restorations, were fitted with steel threads and reused, retaining their heads. Silicone rubber was used to make a copy of the metal parts, which were then used to make a plaster copy of the doors (now in the church of San Francesco alla Scarpa, the current location of the Soprintendenza of Bari) [18]. The door was finally placed inside the church to protect it from the aggressive marine microclimate. Today, a copper alloy copy of the doors is placed at the original location of the doors.

Three of the lowest four panels of the door from Monreale were substituted at the end of the 17th/beginning of the 18th century. They show the coat of arms of cardinal Roano from Monreale (1673–1703) [4]. Additionally, the current wooden base derives from the same period. It is made of mainly softwood (silver fir; *Abies pectinata*) and some chestnut wood. Moreover, many of the original nails were also substituted when the door was completely unmounted and the new panels were applied. After this intervention, at an unknown time, the lower parts of the wooden structure were substituted with new pieces. In 1974, the ICR took some patina and metal drilling samples for analysis (unpublished report; see [19]). Chemical analyses of metal samples from some frame elements were carried out; from the panels themselves, only patina samples were taken. Metallographic analyses of two frame elements revealed a recrystallized grain structure with an (α+δ) eutectoid of the Cu–Sn system. In 1983, restoration work was carried out by Ignazio Di Bella [19] without removing panels from wooden support. The restoration of the wooden structure included the removal of dust and dirt, disinfestation with Xylamon and a protective and strengthening treatment with acrylic resins on both sides (as much as possible). The restoration of the bronze parts included a) degreasing of the surface of all the metal parts, carried out with solvents and a basic solution applied in pads; b) sealing of all the spaces and cracks between the metal parts with water-repellent rubber so as not to wet the wooden support (subsequently removed); c) mechanical removal of the upper encrusted layers of corrosion; d) washing of the bronze surface with deionized water; e) stabilizing treatment with a 2% aqueous solution of sodium sesquicarbonate; f) final treatment with deionized water until a conductivity of approximately 15 microsiemens is obtained; g) dehydration of the surface after each washing with jets of hot air; h) treatment with benzotriazole; and i) application of a protective film and a microcrystalline wax. Notably, reproduction of the lower left rosetta next to panel A7 also occurred [19]. Despite this work, 40 years later, the doors were again in a very poor state. As a result, further restoration work was needed. Restoration of the door started in October 2022 right after the author's on-site studies and was completed in July 2023 under the supervision of the Superintendence (soprintendenza) for the Cultural and Environmental Heritage of Palermo.

## 3. Methodology

### 3.1 Photographic documentation

During our investigations, thorough photographic documentation of the doors was conducted to support the subsequent analyses. This process followed a methodological framework and workflow similar to that utilized for the bronze doors of San Marco, Venice, and San Zeno, Verona [20,21]. The main aim was to establish a precise metric foundation by creating a high-resolution digital surface model of the relief front side along with a corresponding high-resolution true orthomosaic. In addition to capturing high-resolution images of individual panels and areas of interest, the front side of the doors was documented via image-based modeling techniques. Image-based modeling employs photogrammetry to reconstruct three-dimensional surfaces from a series of 2D photographs taken from various camera angles [22]. The versatility and usability of derived 3D models and calculated orthoimages are evident and well acknowledged in the field of cultural heritage documentation [23]. To address the research questions concerning the investigated objects, the photographic documentation aimed to provide a data basis contributing to the questions of the chronology and the construction of the doors.

The scalability and robustness of the method and equipment facilitated the seamless integration of photographic documentation alongside other data collection procedures. The data capture was conducted concurrently with chemical analyses and observational descriptions of structures and details in situ. Owing to the different dimensions of the three objects recorded and their different spatial positions, two doors are still installed in functional portals today, whereas one door has been preserved and mounted in the interior of the church building for viewing, the approaches vary slightly.

All photographs were taken using a Ricoh GR IIIx camera equipped with a 26.1 mm focal length (57° diagonal angle of view) and an RGB primary color CMOS sensor (23.5 mm × 15.6 mm, 24.24 megapixels, 3.9 µm pixel pitch). For data acquisition in Monreale and in Ravello, the camera was used on a gimbal mounted on a 3 m monopod for the upper parts. The lower parts were photographed handheld without an additional extension pole. Owing to their position outdoors, the illumination provided by dispersed natural light could be utilized while avoiding direct sunlight on the object. For the door in Ravello, a scaffolding had to be used in combination with the pole-mounted gimbal for the upper parts. The location of the Trani door within a dark lit church room required additional artificial lighting, which was realized by two 100 W LED spotlights with a 5600k color temperature.

The images were processed via Agisoft Metashape software (version 1.7.2). Scale was established through measurements of specific distances on the object, and scaled poles (horizontal and vertical; meter sticks 2 and 3 m in length with mm marking, CE accuracy class II/error within 0,035%) were included in the acquired photographs to serve as reference scale bars during processing.

The dataset obtained for the door of Trani (4.92 m X 2.76 m) was generated via 1,388 camera stations with an average perpendicular distance of 1.48 m to the object. The reconstructed surface yielded a sampling distance on the object of 0.216 mm per pixel. The calculated scaling error should be within 0.0938%, controlled by the scalebars with a measured distance of 2.5 m. The data for Ravello (3.78x2.66 m) are based on the processing of 1,188 camera stations to a reconstructed surface with 0.223 mm per pixel. The control measurement gives an error of 0.0225% at the scale bar of 2 m. For the side door of Monreale, 541 images were used, and a surface with a resolution of 0.206 mm per pixel was achieved. The error when a scale bar measurement of 2 m length is used is within 0.1047%. To generate products for further analyses, it was necessary to define a projection plane for every single door. This was achieved by the definition of a plane, providing a local rectilinear coordinate system, by the definition of a horizontal and vertical axis on door parts with equal relief levels. This plane was used as a projection plane for the generation of three Digital Surface Model (DSM) with a resolution of 0.2 mm per pixel. Based on that a true orthophoto orthomosaic was generated at a resolution of 0.2 mm per pixel for each object.

In addition to these data products, which depict the entire objects (with the exception of areas visually concealed by portal elements at the edge), defined object areas were selected for a detailed comparison for all three doors. For certain panels that represent existing motifs on all three doors, additional photographs were taken, which enabled an additional increase in the resolution of the calculated areas through close-ups and were to be used for direct comparison. The processing steps for these subsamples were the same as those for the full-coverage datasets but yielded higher-resolution models for future detailed comparisons and analyses.

The individual DSMs were visualized and processed in a geographic information system (GIS, QGIS 3.28) to answer the questions posed in the article. For visual and metric analyses, relief representations were generated on the basis of a combination of different visualizations of surface data (sky-view factor, hillshade, slope gradient, and openness). This toolset was developed for the visualization and investigation of raster data at a landscape scale [24], but the algorithms scale well for the processing of the highest resolution surfaces available within this research. They enable realistic and legible visualization of larger relief structures while maintaining small surface details. In combination/alternation with the high-resolution orthomosaic, elements can be identified that can be used for metric comparisons in the projection plane through measurements of the image.

The extensive data collected during the investigation were managed within a geospatial database in the GIS environment. This consolidated dataset includes tabular data from chemical analyses, geometric data from three-dimensional (3D) modeling, and detailed images of the individual doors, with a focus on their artistic and constructional features. These datasets were systematically organized and combined for analytical purposes.

Subsequent processing stages involved constructing a textured 3D model from a point cloud containing a high point count. Medium-resolution 3D models were exported and uploaded to a data repository to provide them to a broader public Models S1–S3 in S1 File. However, as they are sole byproducts the workflow did not focus on the completeness of the minute surface. Therefore, these at this stage they are not provided for scholarly research but can be generated for such a purpose in future.

### 3.2 Chemical analyses and principal component analyses

The **chemical composition** of the metal parts was determined via an Oxford Instruments portable ED-XRF analyzer (model: X-MET5100) with a high-resolution detector and a 45 kV Rh target X-ray tube. The analyzer operated at a voltage of 40 kV, current of 10 µ A, and acquisition time of 60 s. Spot measurements were conducted with a diameter of approximately 9 mm. The quantitative detection of alloying elements such as Cu, Sn, Zn, Pb and Sb (only in the case of Monreale) was achieved, whereas other elements were detected qualitatively because of the presence of surface corrosion layers. Sulfur (S) could not be detected by this ED-XRF analyzer. Calibration standards for alloys with different chemical compositions were used [20]. Each metal panel of the doors was analyzed in 5–10 different areas to determine the mean chemical composition (S1 Table). However, owing to the heterogeneity of the composition of the panels in different areas due to the casting process and different corrosion processes, an average result for each element was not calculated, despite an adequate number of analysis points. Chemical analyses were carried out on all the panels of the three doors, except for the top three rows of Trani, as we had no scaffolding, only a ladder, and it was simply not possible to reach the top panels with the portable ED-XRF analyzer. The chemical results obtained for the doors of Monreale are supported by chemical analyses by SEM-EDXS carried out by some of the authors (M.M.; G.G.) on metallographic and drilling samples for the Superintendence (soprintendenza) for Cultural and Environmental Heritage of Palermo (soon to be published).

A **Principal Component Analysis (PCA)** was performed on the data matrix following the protocol specified by Mödlinger *et al*. [20]. The main objective of this process is to extract the maximum amount of information from the multivariate data structure by representing it as a combination of variables. PCA involves transforming the original dataset into a new geometric space, where the x-axis represents the first principal component (PC1), which corresponds to the greatest variance in the data matrix. The second principal component (PC2) is represented on the y-axis, perpendicular to PC1, indicating the next highest variance in the data. PCA produces two graphical representations: i) loadings and ii) scores. Loadings are shown as a matrix, where each column represents the eigenvectors and each row represents the original variables. The significance of each original variable within that eigenvector is indicated by the numerical coefficients in each row. Similarly, the scores are presented as a matrix, with rows representing the samples and columns indicating the principal components. The score matrix corresponds to the projections of the samples in the newly defined space. The computations were performed on the centered and autoscaled raw data matrix via the Chemometric Agile Tool (CAT) software [25].

## 4. Results and discussion

One of the most important questions of art history is the chronology of the three doors that are preserved today. The Ravello and Monreale doors can be dated by the donor inscription and the construction of the Monreale Cathedral, but it is still unclear when the Trani door was built. According to the outdated, developmental narrative argument, the Ravello door would have been made before the Trani door because the Trani door has more ornamentation [1,2,4,10]. New approaches also consider workshop processes. It is striking that the Ravello door reuses a panel from Trani and complements it. The kneeling angles that form the round-arched finial of the Trani door also appear in the upper register of Ravello (S1 and S2 Figs). However, as the Ravello door is rectangular, the angel panels were supplemented by a triangular segment in which Sol and Luna now appear. On this basis, the order of the doors would then be Trani (prior to 1179), Ravello (1179), and Monreale (approximately 1185/86, taking into account the construction history of the cathedral). However, looking at the chemical composition of the metal parts and the way they are made, the situation becomes more complicated. The panels on the three doors clearly show that most of the panels are exactly the same as those on the other doors (Table 1). This begs the question of how they could be so similar:

**Table 1:** Overview of the panels present on the four doors of Barisano.

panel	Trani	Ravello	Monreale	Bari
Andrew	1	1	1	1
Angel (facing right)	1	2		2
Angel (facing left)	1	2		2
Archer A		3	1	
Archer B	3			
Bacchus			1	
Bartholomew	1	1	1	1
Christ Pantocrator			2	
Coat of arms Roano			2	
Dedicatory Iscription		1		
Deposition	1	2	1	1
Door Handle	2	2	2	2
Elijah	1	2	1	1
Eustace	1	2	1	
Eustace	1	2	1	
George	1	2	1	
Harrowing of Hell	1	2	1	1
James the Greater	1	1	1	
James the Less		1	1	1
John Ev.	1	1	1	1
John the Baptist	1	1	1	1
Maiestas Domini	2	2		1
Matthew		1	1	1
Nicholas with donor		2	1	2
Nicolaus Peregrinus, Barisanus	1			
Paul	1	1	1	
Peter	1	1	1	1
Philip	1	1	1	1
Simon	1	1	1	1
Thaddeus	1	1	1	1
Thomas	1	2	1	1
Tree of Life	3	10		1
Virgin with Child	1	2	1	1

- Were the same molds used to produce the exact same motif on the panels of the different doors?
- Were the same models used for the production of similar panels [4]?
- Were the motifs copied from one door to create the model for the next door?
- Was there another way of reproducing the panels from one door for the other doors [26]?

In the following, we aim to clarify the different production steps and the chronological order of the production of Barisanus’s doors from Trani, Ravello, and Monreale.

### 4.1 Chemical analyses and principal component analyses (PCA)

#### 4.1.1 Chemical analyses.

Initially, a univariate approach was used to determine the average composition of the doors. All three surviving doors were made of leaded bronze, i.e., with ≥ 1 wt.% Pb. The different states of preservation, particularly in terms of corrosion, made comparison difficult. Consequently, we will focus on the alloying elements only: Cu, Sn, Pb, Zn, and Sb (for Monreale only); concerning the PCA, these elements are considered quantitative. Other elements, such as As, Ni, or Ag, were considered qualitative and only present or absent.

**Fig 3:**
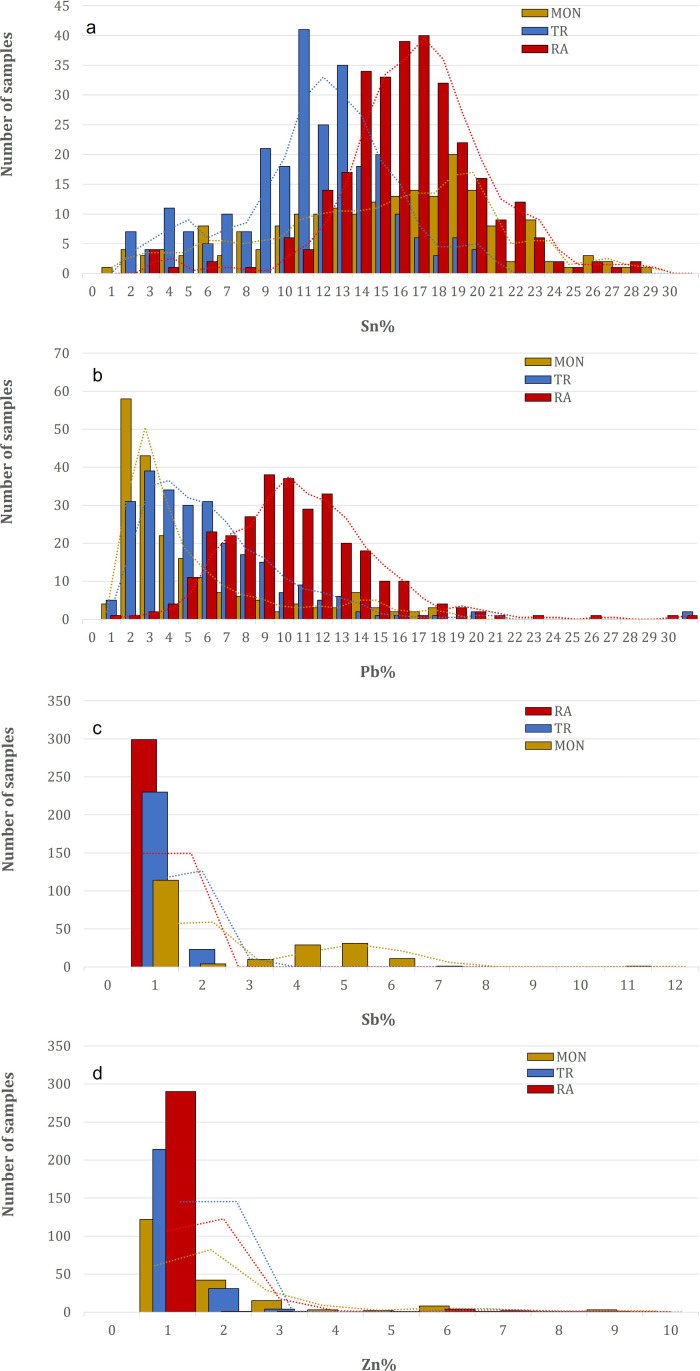
Frequency distributions of the main alloying elements from the XRF analyses of the three doors analyzed (MON=Monreale; TR=Trani; RA=Ravello): a) Sn; b) Pb; c) Sb; d) Zn.

There are significant differences in chemical composition between the doors: while the doors from Trani contain between 2–20 wt.% Sn, with the majority of the points analyzed showing a content of 11–13 wt. %, the doors from Ravello contain much more Sn, between 2–22 wt.%, with a unimodal frequency distribution peaking at 14–19 wt.%, whereas the door from Monreale contains approximately 2–25 wt.% Sn (Fig 3a), showing a heterogeneous composition, which is probably related to a thicker and more mixed corrosion pattern. The amount of Pb (Fig 3b) in the alloy also differs from door to door: Monreale has approximately 1–5 wt. %, the lowest amount of Pb for most of the analyses, partially overlapping with Trani, which has a composition between 1–8 wt. %. Some exceptions are noted in the frequency distribution plot for both doors. Ravello is clearly distinguished by the average Pb content, with a unimodal distribution centered at approximately 9–12 wt.%. A particular case is the doors from Monreale: most of the original panels associated with Barisanus contain significant amounts of Sb of up to 7 wt.%, as shown by the bimodal distribution in Fig 3c, with a peak at approximately 4–5 wt.%. Some of the panels, frames, or rosetta also contain some Zn, which does not necessarily point to a more recent reproduction of these elements, except for some points of analysis, which are mostly from the Monreale door and are clearly visible in Fig 3d.

#### 4.2.2 PCA.

A first computational analysis was performed on the entire set of data arranged in a matrix of 761 points of analyses in the rows and 8 columns for the alloying elements. S5a Fig, supplementary material shows the biplot of the analyses of the three doors: PC1 vs PC2, with a total explained variance of 52,3%. The doors can be grouped into two macroclusters, which differ in composition, in accordance with the first analytical results.

The lower cluster includes both the Trani and Ravello doors, which are colored blue and red, respectively, and some points of the Monreale door, which are colored dark yellow. Its composition is clearly different from that of the others. As shown in S5a Fig, partial overlap occurs along the Cu‒Sn direction. Sn shows considerable variation for all doors and is not a discriminant in the PCA. These differences are due to the amounts of Pb and Sb, which are inhomogeneous, as they are visible from some points that are distributed far from the main cloud. In particular, the amount of Sb in the alloy appears to be much greater for the cluster that makes up the Monreale door, which is located in the upper part of the graph. The graph shows a few points outside the main cloud. One of these points, belonging to the Ravello door, is particularly noteworthy and is located in the upper left quadrant (iron_D9-RA) and corresponds to a decorative iron element. It has been identified as an anomaly and therefore ruled out.

A new PCA is then performed ( S5b Fig). In the calculation of the principal components, the two variables Ag and As were also excluded, as they slightly affect the variance and the information carried. The biplot PC1 vs. PC2, with a total explained variance of 53.6%, shows less clear chemical differentiation, related only to the concentrations of Sb and Pb. Other anomalies were noted: Monreale points Ro_blB2, Ro_BlC2, and Ro_BlD1, which all correspond to a decorative element (rosetta) located on the lower left (Bl) of panels B2, C2 and D1, which have high amounts of Fe ranging from 5 to 10 wt.%. This is the result of contamination by iron door nails, which led to the deposition of rust on the surface by leaching. These points were therefore excluded from the analysis.

**Fig 4:**
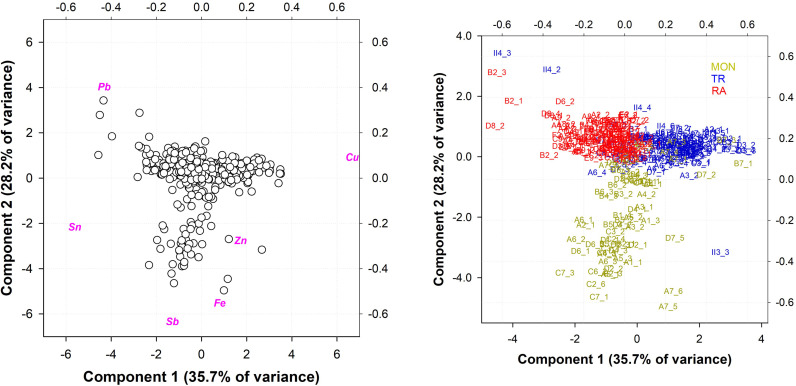
PCA computation on the panels of the three doors: MON=Monreale; TR=Trani; RA=Ravello. a) Biplot; b) score plot. PC1 vs. PC2 explained 62.5% of the total variance.

A more systematic analysis was then carried out, focusing only on the panels that make up all the doors, to further detail any compositional anomalies or similarities. Fig 4 shows the results of the computational PCA of the panels.

**Fig 5:**
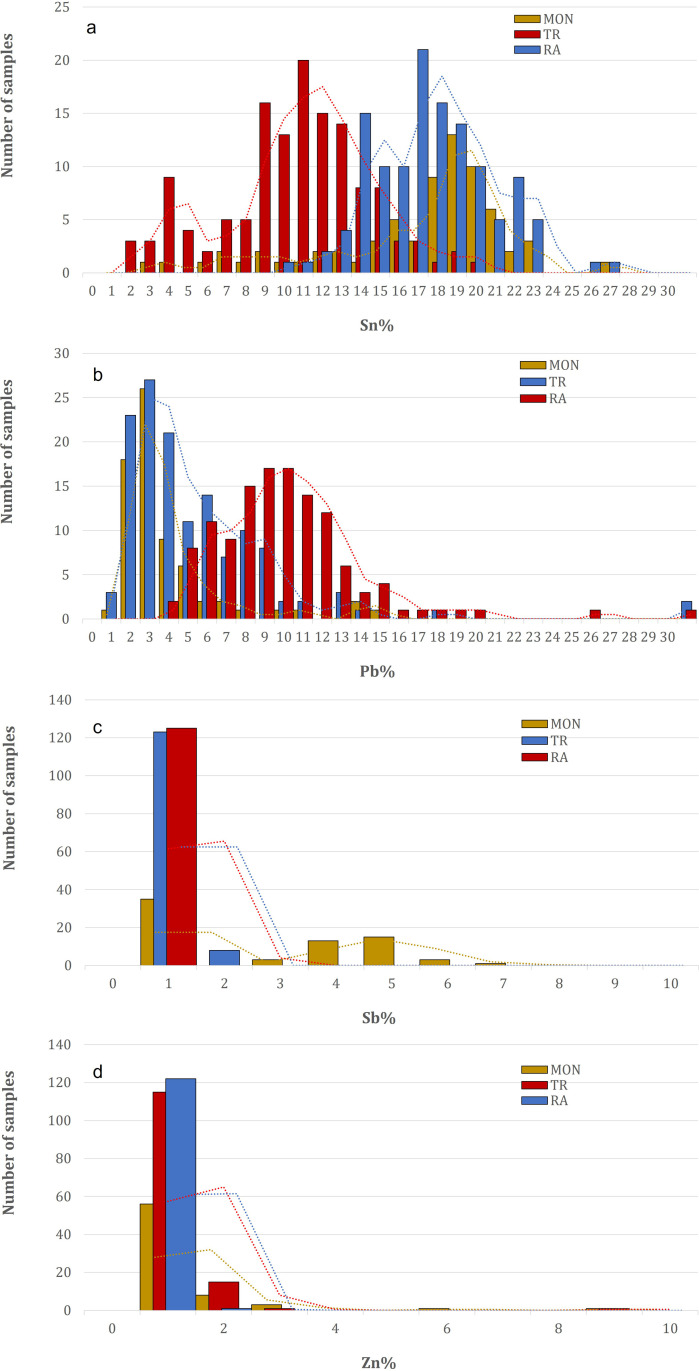
Frequency distribution of the main alloying elements from the XRF analyses of the panels of the three doors analyzed (MON=Monreale; TR=Trani; RA=Ravello): a) Sn; b) Pb; c) Sb; d) Zn.

Excluding the anomalies and the points with superficial heterogeneity due to the variable thickness of the patina, three clusters with a defined range of coordinates can be distinguished, with each cluster mostly coinciding with a door. However, some points do not have the general composition of the door and therefore need to be discussed. Panels A7, B7 and D7 of the Monreale door belong to the upper right cluster and not to the lower cluster (Fig 4**, right**), which describes most of the door’s composition. These panels are clearly of modern manufacture; they were added during the 17th century (see above), with a Zn content above the average composition of the door between 2 and 3 wt. % vs < 1 wt. % and a null Sb content < 1 wt. %), with the highest Zn composition for points A7_5 and A7_6 (8-12 wt.%). When a variable at a time corresponds to a single element, each door has a specific average panel composition (Fig 5), which remains consistent with what has been previously observed.

However, certain points are situated at considerable distances from the typical compositional range of the doors:

- Analyses of panel II4 (points II4_2, II4_3, II4_4 and II4_6) from the Trani door demonstrate a composition consistent with the upper left cluster, which encompasses the majority of the Ravello door. Although the alloy differs from that of the Ravello door, the high Pb content (ranging from 14 to 42 wt. %) leads to an erroneous attribution to the upper left cluster.
- Analysis 3 on panel II3 (point II3_3) of the Trani door reveals a Zn content that does not align with the general composition of the door, which is 8.5 wt. %, even closer to the upper right cluster. Upon closer examination of the points of analysis, it became evident that a visual discontinuity existed, which was likely the result of a repair that was conducted during the early 20th century restorations (see above, section 2**: State of the Art**).
- Analysis 5 and 6 of panel A7 demonstrate a high Fe content (3 wt.%), which categorizes them in the lower right quadrant (Fig 4**, left**). Notably, a high Ag content is observed (between 2 and 12 wt. %, not visible in the PCA), which may be related to a silvering procedure.

**Fig 6:**
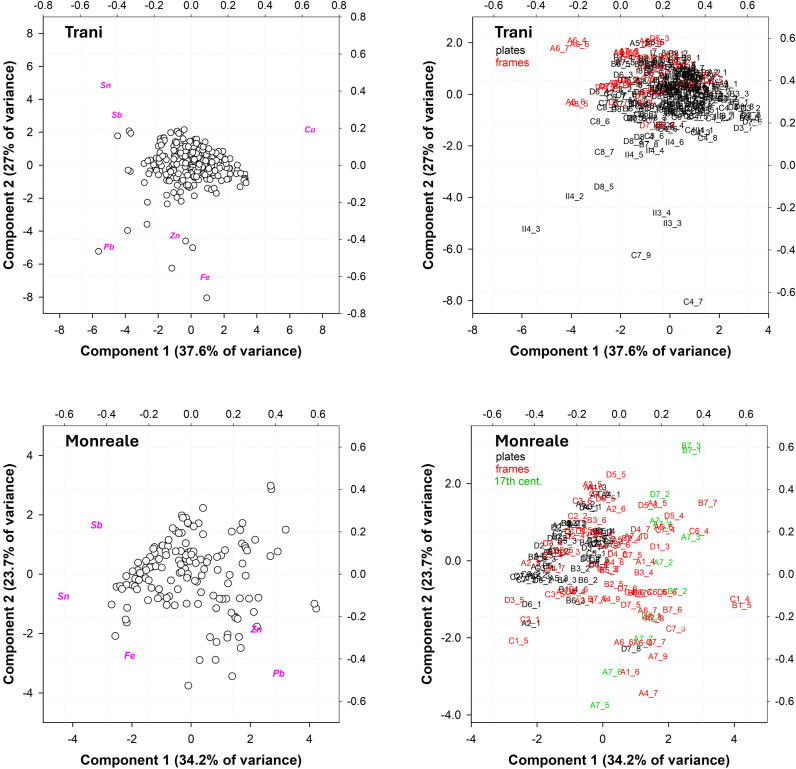
PCA computation of the XRF analyses of the Trani (a and b) and Monreale doors (c and d). a) biplot Trani; b) score plot Trani. The red points in panels A7, A8, D5, D7 and II6 represent samples with relatively high Sb contents. PC1 vs PC2 explained 64.6% of the total variance. c) and d): PCA computation of the XRF analyses of panels (black) and frames (red) at the Monreale door: a) biplot Monreale; b) score plot Monreale. The green dots are the modern panels from the 17th century. PC1 vs PC2 explained 57.9% of the total variance.

**Fig 7:**
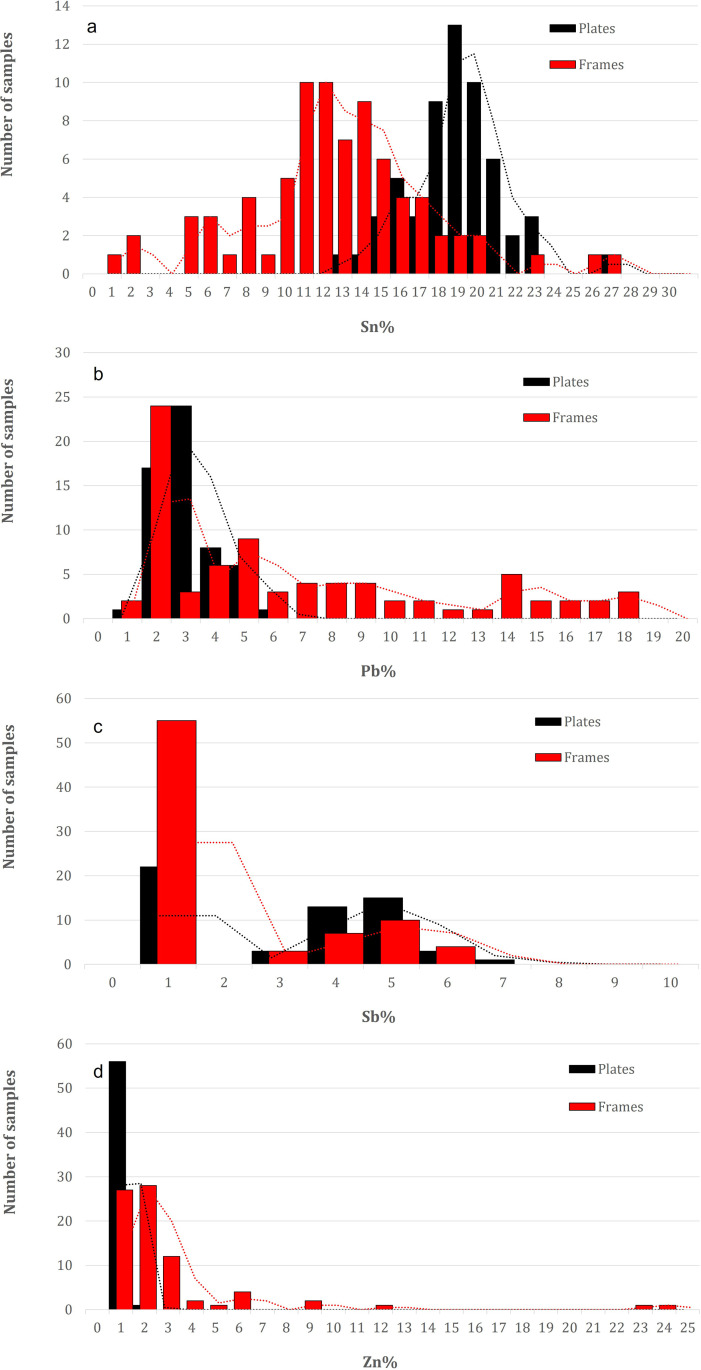
Frequency distributions of the main alloying elements from the X-ray fluorescence (XRF) analyses of the panels (black) and frames (red) of the Monreale door: a) Sn; b) Pb; c) Sb; d) Zn.

**Fig 8:**
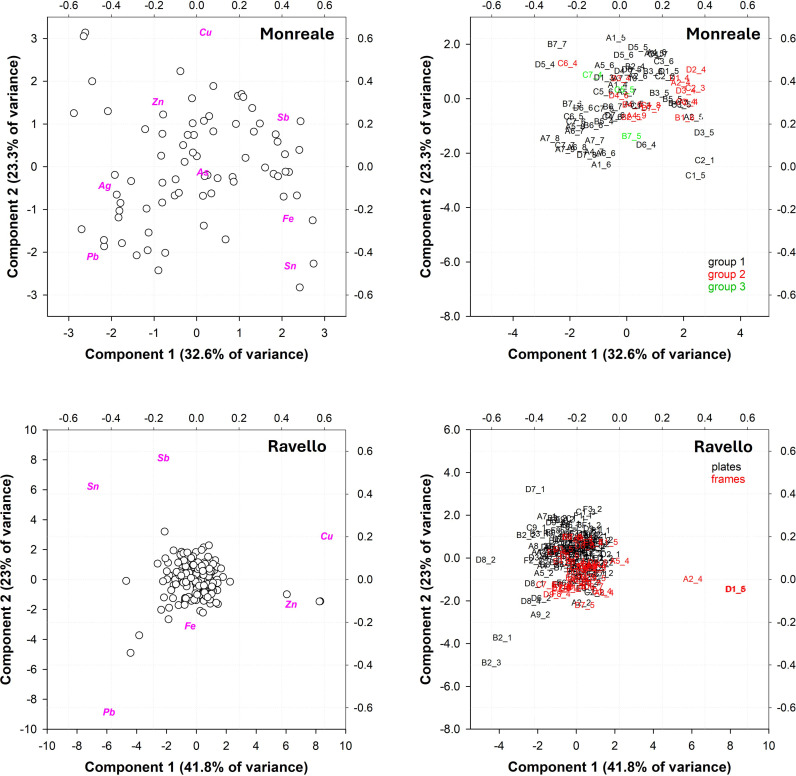
PCA computation of the XRF analysis of frames on the Monreale door according to stylistic interpretation (a and b): a) biplot Monreale; b) score plot Monreale. PC1 vs PC2 explained 55.9% of the total variance. C) and d), PCA computation of the XRF analyses of panels (black) and frames (red) on the Ravello door: c) biplot Ravello; d) score plot Ravello. PC1 vs PC2 explained 64.8% of the total variance.

**Fig 9:**
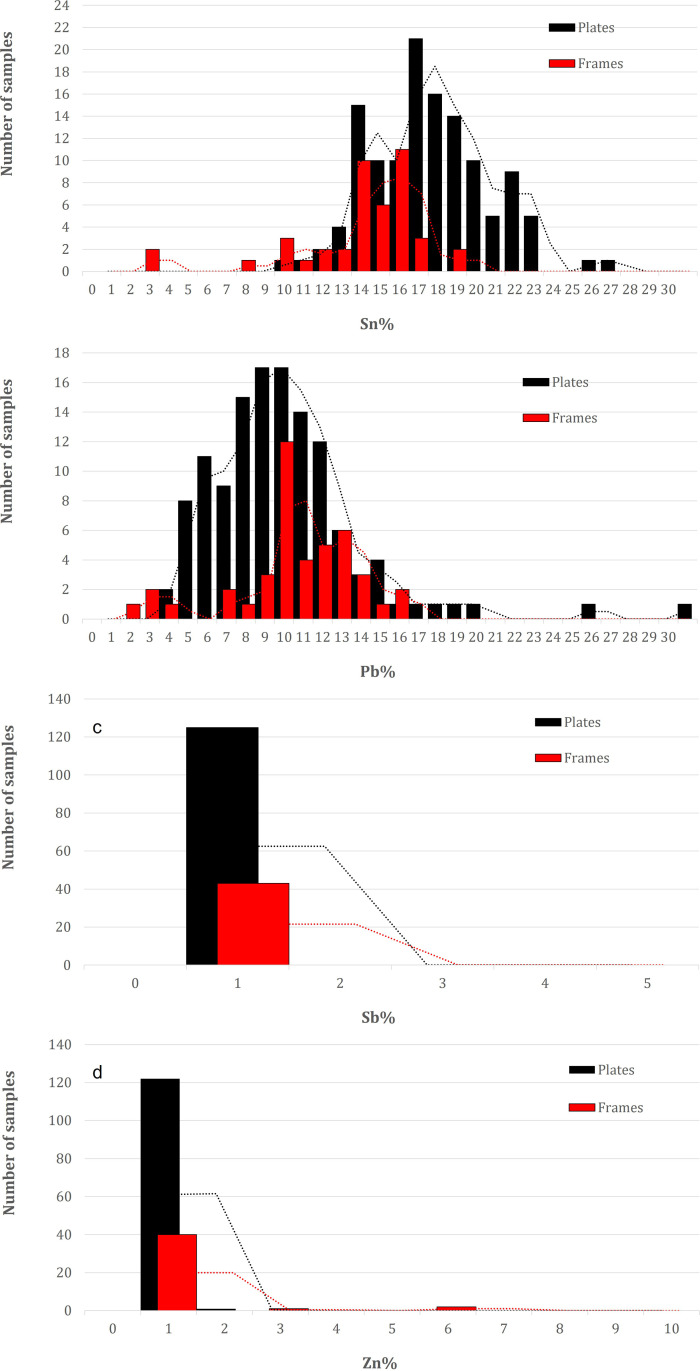
Frequency distributions of the main alloying elements from the X-ray fluorescence (XRF) analyses of the panels (black) and frames (red) of the Ravello door: a) Sn; b) Pb; c) Sb; d) Zn.

**Fig 10:**
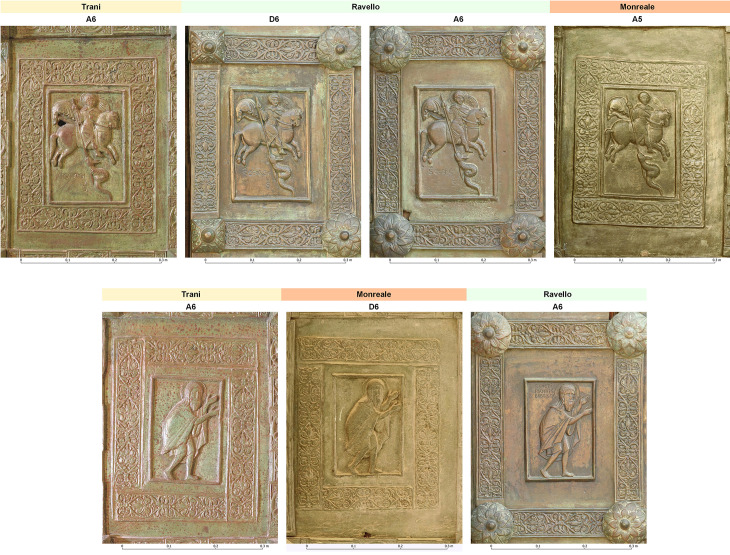
Top: Panels from St. George. From left to right, the motif panels are consecutively smaller, i.e., Trani was cast first, then the two panels of Ravello; finally, they were presumably copied from Ravello for the Monreale doors. The panels of Ravello are identical in size, indicating that their wax model was made at the same time with the same mold (© M. Fera/Novetus/GAPAMET). Below: Panels from John the Baptist. The dimensions decrease from left to right. Trani was made first, then Monreale, after which Ravello. In this case, the sequence is different than that of St. George, suggesting that, at the very least, the wax models for Ravello and Monreale were made more or less contemporaneously (© M. Fera/Novetus/GAPAMET).

Once it was established that the single doors exhibited a slight but statistically significant compositional difference, further analyses were conducted on the single doors, focusing on the decorative elements, namely, the frames and panels for the Monreale and Ravello doors, to assess potential compositional consistency. For the Trani door, we did not differentiate between frames and panels, as they were cast as one unit. The PCA elaboration of each single door is shown in Figs 6 to 11.

Upon examination of the elaboration performed on the **Trani** door (Fig 6a and b), it becomes evident that a main cloud is present at the center of the graph, indicating that the elemental composition of the door is highly consistent. Some points, whose coordinates are distant from the main cloud, require further examination, as they clearly distinguish the contents of Sb (upper left quadrant) or Pb, Zn and Fe (lower quadrants). Panel A6 (analyses in red in Fig 6a and b) shows a higher Sb content, approximately 1.5–2 wt.%, than the average alloy composition (<< 1 wt. %) utilized for the door. Furthermore, panels A7, A8, D5, D7 and II6 present the same elemental content indicated in red in both the biplot and the score plot in Fig 6a and 6b, which may indicate the utilization of a distinct alloy that is compositionally compatible with the Monreale door (visible also from Fig 4) for the fabrication of these scenes. Nevertheless, it is not possible to ascertain any chronological implications related to a change in alloy supply.

Some points located in the lower quadrants (II and III) were previously discussed (II4_2 and II4_3, high Pb content; II3_3, repair). Additionally, several other points were identified: the II3_4 point is also a repair (with the same Zn content as II3_3); the C7_9 point is a modern decorative element, a rosette, with a Zn content of approximately 23 wt.%, which is incompatible with the ancient alloy composition; and the C4_7 point is an outlier, as an anomalous Fe content of 6 wt.% is related to contamination from iron nails.

Fig 6c and d display the PCA performed on panels and frames of the **Monreale** door. The points of analysis are arranged in two main clusters and along an imaginary line that connects Pb to Sn (component 2). Two distinct compositions can be identified according to the percentage of elements present. The cluster on the left is characterized by a higher concentration of Sn and a lower concentration of Pb, and vice versa. The majority of the points belonging to the left cluster are derived from panels, and a similar assumption can be made for the right cluster (frames). The frequency distribution plot (Fig 7) indicates that there is a statistically significant difference in the average composition of the panels and frames.

This finding is consistent with the PCA results, which indicate that the Sn content in the panels is higher on average, with a unimodal distribution peaking at 18–20 wt.%, than in the frames, with a bimodal distribution peaking at 11–12 and 15 wt.%, suggesting a slight distinction in the composition of the alloys used. Furthermore, a partial overlap of compositions is observed in the frequency distribution, as evidenced by the partial overlap of the red points representing the frames, which are situated within the left cluster and thus exhibit a composition similar to that of the panels. Additionally, modern panels produced in the 17th century are plotted (green points of analyses in Fig 6c and d). In addition to a stylistic difference in appearance, they clearly exhibit a composition that is more closely aligned with the right cluster (frames).

Furthermore, we sought to ascertain whether a compositional correlation exists between frames bearing the same decorative motifs. A PCA on all frames of the Monreale door is presented in Fig 8a and b. The plots presented here represent three different groups of frames according to the stylistic interpretation of [1].

The majority of the frames in groups 1 (black), 2 (red) and 3 (green) exhibit a stochastic arrangement in the graph, derived from a varying chemical composition. The samples can be divided into two subgroups on the basis of their Sb content. However, the actual differences in Sb are relatively minor, and the distinction is an artifact of the elaboration process. Exceptions are the frames of groups 2 C1_4 and C6_4, which exhibit markedly lower Sn contents of 2 to 5 wt.% versus 14 wt.% on average for group 2, and are produced from an alloy more akin to those employed in the other groups. It is not possible to ascertain whether these frames were produced from a different alloy or were the result of remelting. However, given the information currently available, both hypotheses appear plausible.

The PCA elaboration of the panels and frames of the **Ravello** door is displayed in Fig 8c and d. The points of analysis appear to be distributed around a main cloud in the center of the graph, with some anomalies. With values of more than 25 wt. %, points B2_1 and B2_3 clearly have very high Pb contents, which are far from the average Pb content of the door and are likely related to heterogeneities of the surface, probably due to a nonuniform corrosion pattern. Upon distinguishing the panels from the frames, it became evident that there was a distribution pattern toward the Sn variable. This was evidenced by the fact that the panels presented a slightly greater amount of the element on average (see frequency distribution plot, Fig 9), although this difference was not statistically significant.

The frames on the left of panel A2 (point A2_4) and on the left of panel D1 (D1_5 and D1_8, two replicates of the same point) have a chemical composition that is significantly different from that of the main cloud. These frame elements, like all the other undecorated flat frame elements, were added in the 19th century. They are composed of a modern quaternary alloy (7 wt. % Sn; 2 wt.% Pb; 3 wt. % Zn). During the last restoration, these frame elements were rearranged and are now mainly found in the upper part of the left wing. The round, undecorated buttons are all from the 19th century.

#### 4.3.3 The question of antimony in the doors of Monreale.

The high levels of Sb, up to 7 wt.% by weight, in the panels of Barisanus’ Door at Monreale raise several questions about the origin of this element. In the 12th century, antimony was not yet known as a pure element, so it must have entered the alloy in the form of a mineral, either by using an antimony-rich copper ore or by adding an antimony mineral such as stibnite or antimony(III) sulfide (Sb_2_S_3_) to the melt or by cosmelting both types of ore.

However, single objects made entirely of antimony, or high-antimony copper alloys, are already known from prehistory. The earliest Sb-rich artifacts are known from Egypt: a potential vase made of antimony from approximately 3000 BCE, a copper sheet plated with antimony from 2500-2000 BCE, and from Israel; some of the Nahal Mishmar hoard objects were made of Sb-rich arsenical copper [27]. Pliny the Elder, in his Natural History, described various ways of producing antimony sulfide as early as 77 CE [28], which was used mainly for cosmetics, medicine, and the coloring of glass. However, the earliest description after Pliny was written by Vannoccio Briniguccio in 1540 [29], who noted that stibnite was imported by Venice from Germany to add it to bells, allegedly enriching their sound.

Taking into account contemporary sources about Sicily’s mines—for example, Zakariya al-Qazwini (or ‘Al Qazwîni; 1203-1283) noted that “Sicily has different mines for gold, silver, copper, iron, lead; and they also have alum, antimony, vitriol, ammonia salt and mercury” [30], we can assume that Barisanus (or his workshop), indeed, might have used local metal sources for the production of doors, even though there is yet no documentation about mining activities in the 12th century.

Antimony is a commonly found metal in the mineral deposits of the Peloritani Mountains. It exists in the form of antimonite (Sb_3_S_2_) and is also a component of complex sulfosalts such as tetrahedrite ((Cu,Fe,Zn,Ag)_12_Sb_4_S_13_) and bournonite (PbCuSbS_3_). These minerals are exploited for various metals, particularly copper, when they are found alongside chalcopyrite (CuFeS_2_) and bornite (Cu_5_FeS_4_) [31]. A noteworthy discovery was made at the Selinunte site, where a 3 kg piece of antimony (likely Sb_3_S_2_?) was found [32], indicating early mining activities in the area. Although information about local mining operations prior to the 12th century is unknown, mining for different metals occurred during the 14th century [31]. At Roccalumera, antimonite mineralization associated with antimoniferous and slightly argentiferous galena has also been explored during recent centuries. In this case, the antimony produced contained discrete traces of arsenic and thus was unlikely to have been used for the production of Barisanus’ doors.

As we do not have any indications or contemporary descriptions that antimony was added either as a mineral to copper or co-smelted with copper minerals or added in the form of stibnite to copper, it is most likely that Barisanus (or his workshop) used local copper made from Sb-rich copper ores such as bournonite for the casting of the panels at the door of Monreale.

### 4.2 Manufacture of the doors

The digital twins of the doors and the resulting orthophotos provide an excellent basis for accurate, precise and repeatable measurements of the dimensions of the various elements of each of the doors. The orthophotos are free of distortion, allowing us to compare the dimensions of each motif panel with each other. The Barisanus doors offer a unique opportunity to study the same motif in three different doors. Before the preparation of orthophotos of these doors, the following approach would have been infeasible, and now, a multitude of questions concerning foundry practices can be addressed quantitatively. Most importantly, model making and mechanical means of reproducing models with a type of intermediate mold are needed. The method is straightforward. Several measurements of each of the motif panels were taken and subsequently compared. These motif panels were created by employing a mechanical reproduction process because the motif panels themselves are too similar.

If Barisanus used a mother mold to produce all the wax models for all the doors, it is expected that the panels are all of the same dimensions. If the dimensions differ, a more complex model is needed to explain their manufacture.

Solid shrinkage, or volume reduction, can be observed in almost all metals that cool to room temperature after solidification [26]. This phenomenon should not to be confused with liquid shrinkage, which happens in the process of the transition from the liquid to the solid-state and can be compensated for by an adequate gating and feeding system. The solid shrinkage we are concerned with here is the amount of volume reduction that takes place after solidification. It is directly related to the coefficient of expansion. Depending on the geometry of the model and the mold, there may be shrinkage obstacles such as ribs or plungers, which will result in different shrinkage values. To compensate for solid shrinkage, modern foundry practice anticipates this by increasing the dimensions of the casting pattern or casting model. This is called the shrinkage allowance and is specific for each material. Shrinkage is therefore the key to ordering the production sequence of the panels: the smaller the dimensions are, the later they are cast.

In addition to the solid shrinkage of the metal, two other factors should be considered: the dry shrinkage of the mold material and the liquid and solid shrinkage of the bee wax [26]. It suffices here to concentrate on the solid shrinkage of the cast objects, as this presents the minimum of the expected difference in the dimensions of the model and cast. Here, the use of beeswax is assumed, although we know from Theophilus [33] that “adeps” are used for large objects such as bells (for a discussion, see [34]). Adeps may be translated with tallow, and its properties are sufficient if the models are simple, but it does not lend itself to either a warm climate or intricate modeling operations. Both restrictions apply for Barisanus and his workshop.

Table 2 shows the dimensions of two of the ten motifs examined, which are present on all three doors. In some cases, the panels are present twice on the same doors. These methods may yield promising results with respect to the production sequence of the individual motif panels. For ease of comparison, the percentage of the dimensional difference is always calculated in relation to the smaller of the two panels in question. This is in line with modern foundry practice, where shrinkage is expressed in relation to the cast object and not in relation to the dimensions of the model.

**Table 2:** Measured dimensions of the St. George and John Baptist motif panel. The error incorporates the propagated error of the measurement uncertainty of ± 0.2 mm. The motif panels are ordered according to their respective dimensions. The columns to the left present the larger ones.

	Distance in mm				dimensional change in percent								
St George	Trani (T)	Ravello (R)		Monreale (M)	T		R	R		M	R		
	A6	D6	A6	A5	A6	:	A6	A6	:	A5	D6:A6		
lance tip to dragon tail	236.1	231.0	230.9	227.5	2.2	±	0.1	1.5	±	0.1	0.0	±	0.1
hooves	142.6	139.1	138.4	137.8	2.5	±	0.2	0.9	±	0.2	0.5	±	0.2
horse tail to tip front hoove	141.9	139.8	139.3	137.4	1.5	±	0.2	1.7	±	0.2	0.4	±	0.2
	**Trani (T)**	**Monreale (M)**		**Ravello (R)**	**T**		**M**	**M**		**R**			
**John the Baptist**	**A4**	**A1**		**A5**	**A4**	:	**A1**	**A1**	:	**A5**			
calve left -coat left- upper thumb	167.2	160.1		156.2	4.4	±	0.2	2.5	±	0.2			
nimbus right frame- heel right foot	214.9	205.7		201.7	4.5	±	0.1	2.0	±	0.1			
index finger right frame – left foot small toe	186.2	182.1		177.4	2.3	±	0.2	2.6	±	0.2			
nose tip to right ball of the foot	175.6	169.2		165.6	3,8	±	0,2	2,2	±	0.2			

We can see that the Trani motif panels are the largest in both cases. This is also true for the other eight Trani motif panels measured. For further details and a detailed discussion of the expected variability in shrinkage values, see [26]. From a foundry perspective, these are the panels that were cast first. For St. George, the respective dimensional changes suggest the sequence Trani – Ravello – Monreale. Dimensional differences range from 1.5 to 2.5 ± 0.2%. This agrees well with the solid shrinkage values reported for copper-based alloys. The dimensional change from the Ravello doors to the Monreale doors is somewhat smaller but still plausibly explained by solid shrinkage. As St. George appears twice in the Ravello doors, it was possible to compare the dimensions of the panels (Fig 10**, top**). Table 2 shows that these values fall quite well within the range of measurement uncertainty. It can therefore be concluded that they were made from the same model.

Looking at St John, the Baptist panel (Fig 10**, below**), it is immediately clear that the production sequence is different in terms of dimensions Trani–Monreale–Ravello. The dimensional changes are also greater, and it seems plausible that the Monreale panels might not have been cast off the Trani panels directly but were cast off from a door that no longer exists, presumably from the Bari doors. Two explanations seem plausible at this stage:

- The Monreale motif panels were cast off panels, which were made by using the Trani panels and are now lost;
- The Monreale copies were replicated via another method, resulting in greater shrinkage.

The Ravello panels of St John do appear to have been replicated from the Monreale panels.

Looking at just these two panels, it is clear that a simple hypothesis for the production sequence of Barisanus’ doors can no longer be entertained. The other eight panels follow the same pattern. Trani was produced first, followed by Ravello and Monreale, and in half the cases, the Ravello panels were produced before the Monreale panels. In the other half of the panels, the order of Ravello and Monreale is reversed [26].

The workshop used at least two different methods of joining panels and frames. In Trani’s case, the frame elements were part of the panels (i.e., cast together). These ‘frame panels’ were nailed to the wooden support, overlapping in part with the neighboring panels. On the other hand, the panels and frames of the doors at Ravello and Monreale were made independently. The panels were nailed to the wooden support, the frame elements placed over the gaps between the panels were also nailed to the wooden support, and finally, at the corners of the panels where the frame elements meet, large, decorative bosses were nailed to the wooden support to cover the joints of the frame elements [1,13].

## 5. Conclusion

Owing to their precise dimensions matching those of the originals, digital twins offer a robust foundation for thorough investigations into art technology. Distinguishing between originals and casts has become significantly more feasible, as measurements can be effortlessly repeated on digital replicas. Since these observations are grounded in quantifiable data, they can bolster hypotheses that previously relied solely on stylistic considerations. The measurements of the selected panels present on all three doors, which provide information on model and metal shrinkage, confirmed that the Trani door is the oldest of the three surviving Barisanus doors. No historical sources have survived to date the construction of this door, so we must assume a date prior to the Ravello door in 1179.

These measurements also confirm that both the Monreale and Ravello doors were built after the Trani door, as is also suggested by the addition of the spandrels on the four angel panels in the case of Ravello. However, the chronological sequence of Monreale and Ravello could not be clarified: the molds were used interchangeably, as illustrated by the panels of St. George and St. John, the Baptist: in the first case, the Ravello panel was copied to produce the Monreale panel, whereas in the second case, the reverse was true. It seems reasonable to assume contemporary production, if not of the doors, then of the wax models. These wax models could also be easily transported for the production (i.e., casting) of the metal parts of the doors on site. Owing to the high risk of breakage, it is unlikely that the finished molds were transported.

Local casting, i.e., casting next to the church or cathedral, as we know it also from Aachen, is also supported by the different chemical compositions of each of the three doors: contemporary production in the same workshop would inevitably have led to the use of similar alloys. Additionally, most likely, the metal was provided directly from the patron financing the doors. Although all three surviving doors were made from leaded bronze, there are clear differences:

- The Monreale doors were made of leaded bronze with significant amounts of antimony. As we do not have any indications or contemporary descriptions that antimony was added either as a mineral to copper or co-smelted with copper minerals or added in the form of stibnite to copper, it is most likely that Barisanus used local copper made from Sb-rich copper ores such as bournonite for the casting of the panels at the door of Monreale;
- With an average composition of 11–13 wt.%, the Trani doors contain less Sn than do the Ravello doors, which have 14–19 Sn wt.%;
- The Ravello doors clearly contain greater amounts of Pb than the Trani and Monreale doors do, with approximately 9–12 wt.% Pb compared with 1–8 wt.% (Trani) and 1–5 wt.% (Monreale) Pb, respectively.

In short, this study confirms Trani to be the oldest of the surviving doors prior to 1179 and proposes the contemporary production of wax models for both the Ravello and Monreale doors by copying the panels from Trani in a first step and then from Ravello and Monreale in a second step within the same workshop. These wax models were used to prepare the clay molds for the metal parts of Ravello and Monreale via metal provided by a patron. Mounting of the metal parts onto the wooden support then took place directly at Ravello, around the time when it was finished in 1179, and Monreale between 1174, when the construction of the cathedral started, and 1186, when the west portal was completed.

By mid-2025, the high-resolution photographic documentation of both doors and the chemical analyses carried out will be available on the GAPAMET project’s open-access database at https://gapamet.imareal.sbg.ac.at/en

## Supporting information

S1 File Models S1–S3. (DOCX)

S1 Fig Barisanus’ door from Ravello, Campania, Italy. (JPG)

S2 Fig Barisanus’ door from Trani, Puglia, Italy. (JPG)

S3 Fig Barisanus’ door from Monreale, Sicily, Italy. (JPG)

S4 Fig Barisanus’ door from Bari, Puglia, Italy (design of I. Aveta. Reproduction after: BNF, Paris: plate P62820, Vol. VB-132 (A,1)-FOL; © BNF). (JPG)

S5 Fig PCA computation on the matrix of the XRF analyses carried out on the three doors: MON=Monreale; TR=Trani; RA=Ravello. a) Biplot of all decorative elements; b) biplot without anomalies. (JPG)

S1 Table Results of the XRF analysis carried out on Barisanus’ doors from Trani, Ravello and Monreale. (XLSX)

## Permits

All necessary permits were obtained for the described study, which complied with all relevant regulations: Permission to carry out the non-invasive XRF analyses was obtained from the relevant dioceses (Diocesi Amalfi-Cava; Arcidiocesi di Trani Barletta Bisceglie; Arcidiocesi di Monreale) and the responsible local soprintendenze (Soprintendenza Archeologia, belle arti e paesaggio per le province di Salerno e Avellino; Soprintendenza Archeologia, Belle Arti e Paesaggio per le province di Barletta-Andria-Trani; Soprintendenza per i Beni Culturali e Ambientali di Palermo).
