# Supplementary material for: The 12th century bronze doors of Barisanus of Trani in Trani, Ravello and Monreale
Source: PLoS One. 2025 Mar 26;20(3):e0319697. doi: 10.1371/journal.pone.0319697 (PMC11940662; doi:10.1371/journal.pone.0319697)
Supplement: S1 File — (DOCX) [file pone.0319697.s003.docx]

**S1 File.** Model S1. A reduced 3D digital surface model of Barisanus’ doors from Monreale, Sicily, Italy, can be found following this link (Models: M. Fera/Novetus/GAPAMET): <https://sketchfab.com/models/f2cea44702704083803ffdc41571cdb1>. Model S2. A reduced 3D digital surface model of Barisanus’ doors from Ravello, Campania, Italy can be found following this link (Models: M. Fera/Novetus/GAPAMET): <https://sketchfab.com/3d‐models/ravello‐reduced‐2024‐05‐77c5287b0be54f608f4276548f06a569>.

Model S3. A reduced 3D digital surface model of Barisanus’ doors from Trani, Puglia, Italy can be found following this link (Models: M. Fera/Novetus/GAPAMET): <https://sketchfab.com/models/cbc8620db6174f02bc332a8f6e751600>.
