# Supplementary figures and images for: The 12th century bronze doors of Barisanus of Trani in Trani, Ravello and Monreale
Source: PLoS One. 2025 Mar 26;20(3):e0319697. doi: 10.1371/journal.pone.0319697 (PMC11940662; doi:10.1371/journal.pone.0319697)

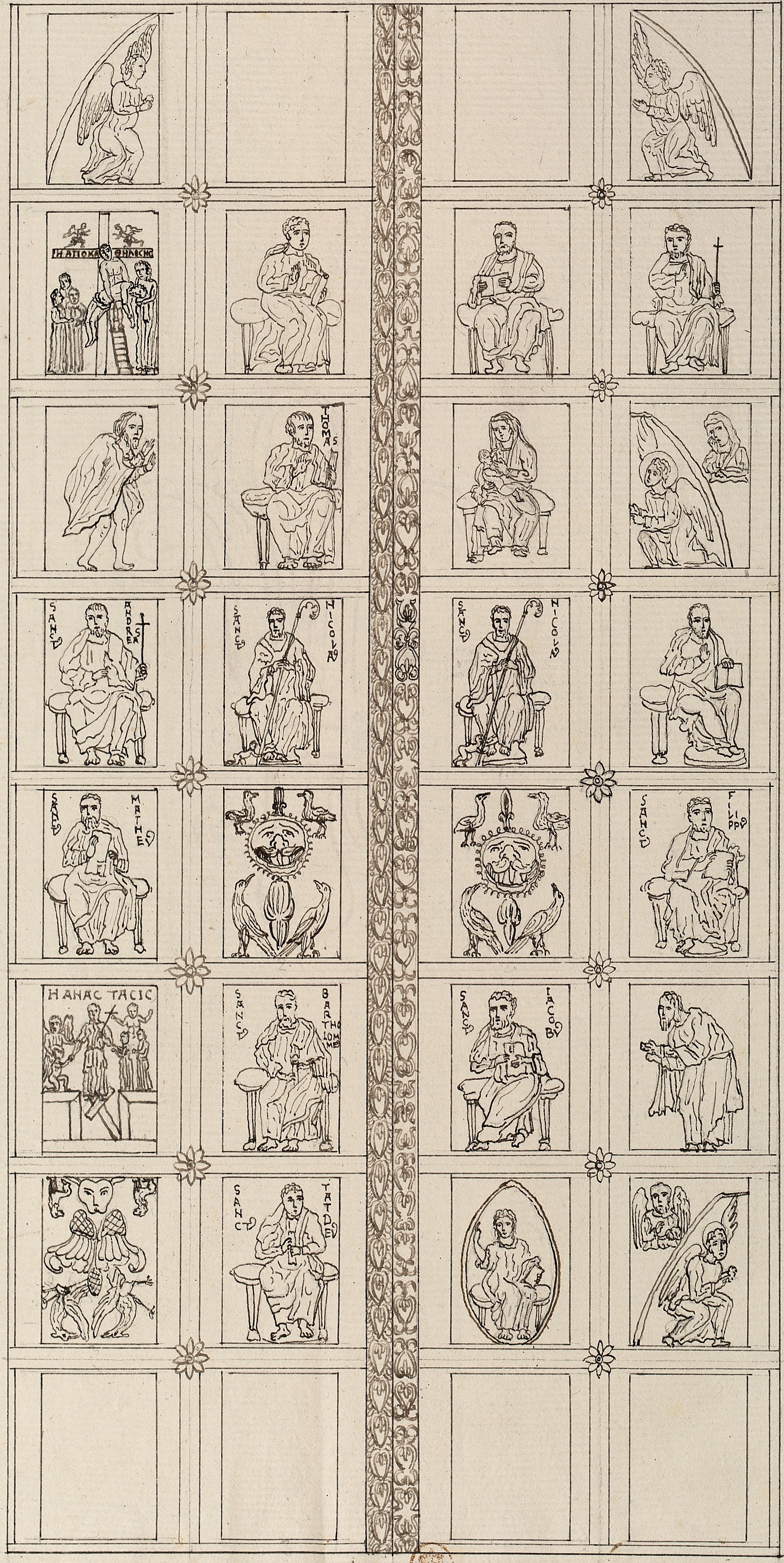

Supplement: S4 Fig — (JPG) [file pone.0319697.s007.jpg]

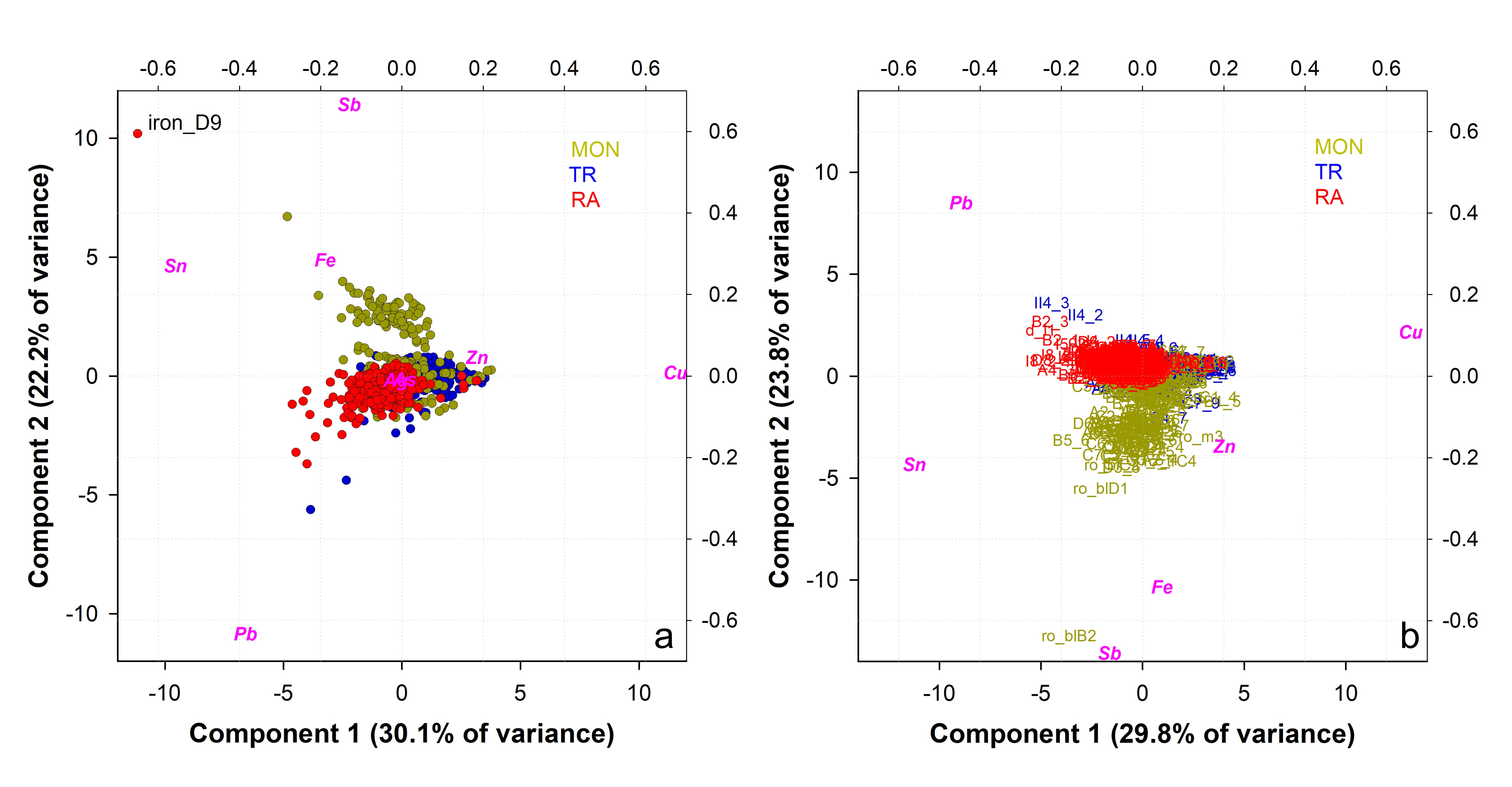

Supplement: S5 Fig — (JPG) [file pone.0319697.s008.jpg]
